# Macrophage-derived interleukin-1beta promotes human breast cancer cell migration and lymphatic adhesion in vitro

**DOI:** 10.1007/s00262-017-2020-0

**Published:** 2017-05-27

**Authors:** Sarah J. Storr, Sabreena Safuan, Narmeen Ahmad, Mohammed El-Refaee, Andrew M. Jackson, Stewart G. Martin

**Affiliations:** 1 Division of Cancer and Stem Cells, Translational and Radiation Biology Research Group, School of Medicine, Academic Clinical Oncology, University of Nottingham, Nottingham University Hospitals NHS Trust, City Hospital Campus, Nottingham, NG5 1PB UK; 2 Present Address: Health Campus, School of Health Sciences, Universiti Sains Malaysia, 16150 Kubang Kerian, Kelantan Malaysia; 3 Division of Cancer and Stem Cells, Host-Tumour Interactions Group, School of Medicine, Academic Clinical Oncology, University of Nottingham, Nottingham University Hospitals NHS Trust, City Hospital Campus, Nottingham, NG5 1PB UK; 4 Medical Biotechnology Department, Genetic Engineering and Biotechnology Research Institute, the City for Scientific Research and Technology Applications, Alexandria, Egypt

**Keywords:** Interleukin-1, Breast cancer, Macrophage, Vascular invasion, Caspase-1

## Abstract

Lymphovascular invasion (LVI), encompassing blood and lymphatic vessel invasion, is an important event in tumourigenesis. Macrophages within the tumour microenvironment are linked to the presence of LVI and angiogenesis. This study investigates the role of macrophage-derived, caspase-1-dependent interleukin-1beta (IL-1β) in an in vitro model of LVI. IL-1β significantly augmented the adhesion and transmigration of breast cancer cell lines MCF7 and MDA-MB-231 across endothelial cell barriers. MDA-MB-231 and MCF7 showed a higher percentage of adhesion to lymphatic endothelial cells than blood endothelial cells following endothelial cell IL-1β stimulation (*P* < 0.001 and *P* < 0.0001, respectively). Supernatants from activated macrophages increased the adhesion of tumour cells to lymphatic and blood endothelium. Secretion of IL-1β was caspase-1 dependent, and treatment with caspase-1 inhibitor reduced IL-1β production by 73% and concomitantly reduced tumour cell adhesion to levels obtained with resting macrophages. Transmigration of MDA-MB-231 cells across blood and lymphatic endothelial monolayers was significantly increased following IL-1β stimulation. Furthermore, supernatants from activated macrophages increased transmigration of MDA-MB-231 cells across endothelial monolayers, which was abolished by caspase-1 inhibition. IL-1β stimulation of tumour cells significantly increased their migratory ability and a significant increase in migration was observed when MDA-MB-231 cells were stimulated with macrophage conditioned media (two of three donors). Results demonstrate that macrophage production of IL-1β plays an important role in the migration of breast cancer cells and their adhesion to, and transmigration across, blood and lymphatic endothelial cells. Results suggest that IL-1β may play a role in the adhesion to lymphatic endothelial cells in particular.

## Introduction

Lymphovascular invasion (LVI), encompassing both blood and lymphatic vessel invasion, is an important event in tumourigenesis and is an initial prerequisite step in metastasis. LVI is strongly associated with adverse patient prognosis in a number of tumour types, with studies showing that in certain tumour types, including breast cancer and melanoma, LVI occurs more frequently in lymphatic vessels as opposed to blood vessels [1–4]. The presence of macrophages within the tumour microenvironment has been linked to the presence of LVI and angiogenesis [1, 5, 6]. Macrophages are recruited by chemotactic factors expressed by tumours and the factors they express can affect tumour cell behaviour; some of the most widely described factors produced by macrophage that can alter angiogenesis are vascular endothelial growth factor (VEGF), and angiopoietin 1 (reviewed in [7]). Certain studies have reported that perivascular macrophages are involved in tumour cell intravasation without concomitant induction of angiogenesis [8].

Despite the link between immune cells within the tumour environment and the presence of LVI only a small number of factors have been identified that may have the ability promote vascular invasion, with most studies focussing upon factors that potentially influence blood vessel invasion. Cytokines are a loose category of small glycoproteins, including interleukins, interferons, chemokines and tumour necrosis factor (TNF). They are produced by a wide range of cells, notably immune cells and also endothelial cells, fibroblasts, and various tumour and stromal cells. A number of cytokines, such as interleukin (IL)-8 and TNF-α, are expressed at high levels by macrophages and can reportedly alter both angiogenesis and metastatic dissemination of tumour cells [9, 10].

The interleukin (IL)-1 superfamily consists of IL-1α, IL-1β and others such as IL-18 and IL-13; and IL1-RA (receptor agonist) which functions to modulate IL-1α and IL-1β activity (reviewed in [11]). IL-1β is a multifunctional pro-inflammatory cytokine principally produced by hematopoietic cells such as macrophages and monocytes, and also by epithelial cells, and expression is associated with angiogenesis and the invasive abilities of tumour cells [12, 13]. IL-1β is linked to increased metastasis in various in vivo models [14] and has been linked with bone-homing in breast cancer. IL-1β expression has been shown to be expressed in the breast tumour microenvironment, at higher levels in invasive breast lesions than benign conditions and ductal in situ carcinoma [13, 15]. Caspase-1, or IL-1β-converting enzyme (ICE), is a cysteine protease responsible for activation of the IL-1β and IL-18 precursors to the pro-inflammatory active cytokines. Studies have suggested that caspase-1 expression is decreased in prostate cancer [16] and colon cancer [17].

This study aimed to investigate whether IL-1β can differentially modulate in vitro tumour cell adhesion and transmigration to, and across, blood and lymphatic endothelium and to assess its ability to alter tumour cell migration. We also sought to determine the effect on these phenotypic endpoints following stimulation with macrophage conditioned media alone and in combination with a caspase-1 inhibitor.

## Materials and methods

### Cell lines and culture

Breast cancer cell lines MCF7 and MDA-MD-231, large vein endothelial cells from human umbilical cords (HUVEC), human microvascular endothelial cells hMEC-1, human telomerase reverse transcriptase immortalised lymphatic endothelial cells (hTERT-LEC) and neonatal dermal lymphatic microvascular endothelial cells (HMVEC-dLy Neo) were used in this study.

Breast cancer cell lines were obtained from the ATCC and used within a 15 passage window. MCF-7 were maintained in RPMI-1640 (Sigma), 10% iron-supplemented donor calf serum (PAA laboratories) with 1% penicillin/streptomycin (Sigma). MDA-MB-231 were maintained in minimal essential medium EAGLE (Sigma), 0.1 mM non-essential amino acids solution (Sigma), 2 mM l-glutamine (Sigma), 1% penicillin/streptomycin and 1% iron-supplemented donor calf serum. HUVEC were isolated as previously described [18, 19] and used between passage 2 and 6. HUVEC were maintained in 37% nutrient mixture F-12 HAM media (Sigma) in sterile water containing 3.7% 199 media (Sigma), 20% iron-supplemented donor calf serum, 1% sodium bicarbonate (Sigma), 14 mM HEPES (Sigma), 2 mM l-glutamine, 1% penicillin/streptomycin, 7.5U/ml heparin (CP Pharmaceuticals), 25 ng/ml epidermal growth factor (EGF) (Peprotech) and 12.5 ng/ml basic fibroblast growth factor (bFGF) (Peprotech). Human microvascular endothelial cells hMEC-1 [20], obtained from ATCC, were grown in endothelial basal medium (Lonza, USA) with 10% iron-supplemented donor calf serum, 1 μg/ml hydrocortisone (Sigma), 10 ng/ml EGF and 1% penicillin/streptomycin and used between passage 4 and 18. hTERT-LEC were a kind gift from Nissato and Pepper [19, 21, 22] and were maintained in endothelial basal media (EBM) supplemented with the EGM-2 bullet kit (Lonza) and used between passage 27 and 34. HMVEC-dLy Neo (Lonza), a primary lymphatic cell line, was cultured in the same medium as hTERT-LEC and used between passage 4 and 6. Cell lines were routinely tested for mycoplasma and tumour cells have been subsequently verified using multiplex short tandem repeat (STR) system (Powerplex 16, Promega).

Tumour-conditioned media were generated from confluent tumour cell monolayers which were then cultured in HUVEC or hTERT-LEC basal medium without serum or growth factors for 24 h. Prior to experimental use, tumour-conditioned media were supplemented with iron-supplemented donor calf serum and 50 U/ml polymyxinB-sulphate (Sigma). Tumour-derived lysate (TDL), used for macrophage stimulation, was generated from 1 × 10^7^ cells subjected to five cycles of freeze–thaw using liquid nitrogen and a 37 °C water bath. Debris was removed by centrifugation and cleared lysates stored at −80 °C.

Healthy donor peripheral blood, obtained with the approval of the relevant ethical review board (BT20052010, University of Nottingham Medical School Ethics Committee), was fractionated using Histopaque 1077 (Sigma) to obtain peripheral blood mononuclear cells (PBMC) [19, 23]. Monocytes were isolated from PBMC using paramagnetic particles conjugated with anti-CD14 antibodies (Miltenyi Biotec) and were >95% pure as determined by flow cytometry. Macrophages were generated by culturing CD14 + monocytes for 6 days in RPMI-1640 medium with 10% foetal calf serum in the presence of 50 ng/ml of macrophage colony stimulating factor (M-CSF) (Peprotech) in Teflon flasks (Thermo Scientific). Macrophage phenotype was confirmed by flow cytometry (CD68+, CD14+, MHC II+) and conditioned media harvested on day 7. IL-1β production by macrophages was induced by stimulating cells with tumour-derived lysate, lipopolysaccharide (LPS) (Invivogen) and a combination of tumour-derived lysate and LPS with and without caspase-1 inhibitor (R&D Systems).

### Static adhesion and migration assays

The assays have been described previously [19]. Briefly, static adhesion assays used a confluent endothelial monolayer that remained unstimulated, or was stimulated with IL-1β for 24 h. Tumour cell adhesion was assessed after 35 min, following cell labelling with 1 μM of Cell Tracker Green CMFDA (Invitrogen). Adherent tumour cells were counted using a fluorescence microscope (Nikon). Two fields of view were counted in each well at 20× magnification. Results were expressed as the percentage of cells adhered relative to control. PBMC adhesion controls were run immediately prior to each experiment to demonstrate that the endothelial cells and cytokine were responding appropriately. In migration assays, a confluent tumour cell monolayer remained unstimulated or was stimulated with IL-1β (5 ng/ml) for 24 h. Mitomycin C (Sigma) was included (10 µg/ml) to inhibit cellular proliferation. Migration was monitored at different time points following a scratch to create an area devoid of adherent cells. Percentage reduction of the scratch area at different time points was measured using ImageJ 1.43u software (National Institute of Health).

### Transmigration assay

A confluent endothelial cell monolayer was grown on Boyden Chamber Transwell inserts and remained unstimulated or was stimulated with IL-1β (10 ng/ml) for 24 h. The confluency and integrity of the endothelial barrier was demonstrated by preventing lucifer yellow leakage (Sigma). Tumour cell transmigration was assessed following cell labelling with 5 nM of Cell Tracker Green CMFDA (Invitrogen). Transmigration was monitored, by counting cells on the underside of the chamber, after 16 h using a fluorescence microscope (Nikon). Experiments were conducted twice, both in duplicate.

### Elisa

Expression of IL-1β in tumour conditioned media and macrophage conditioned media was conducted using a human IL-1β/IL-1F2 duoset ELISA development kit (R&D Systems) according to the manufacturers’ protocols. Briefly, a 96-well plate was coated with capture antibody overnight and the plate was washed and blocked with bovine serum albumin. Samples and IL-1β recombinant protein standard were added to the plate for 2 h. This was followed by detection antibody for 2 h and streptavidin-HRP for 20 min, with each step preceded by a wash. Colour change was achieved by the addition of substrate followed by a stop solution, and the plate was read at 450 nm on a BMG Fluostar Optima (BMG Labtech).

### Statistics

Data analysis was performed in Microsoft Excel 2007 and Graph Pad Prism. Statistical significance was determined using the independent samples *t* test (two tailed), *P* > 0.05 was deemed significant in this study.

## Results

### Tumour cell adhesion to endothelial cells is increased by IL-1β

**Fig. 1:**
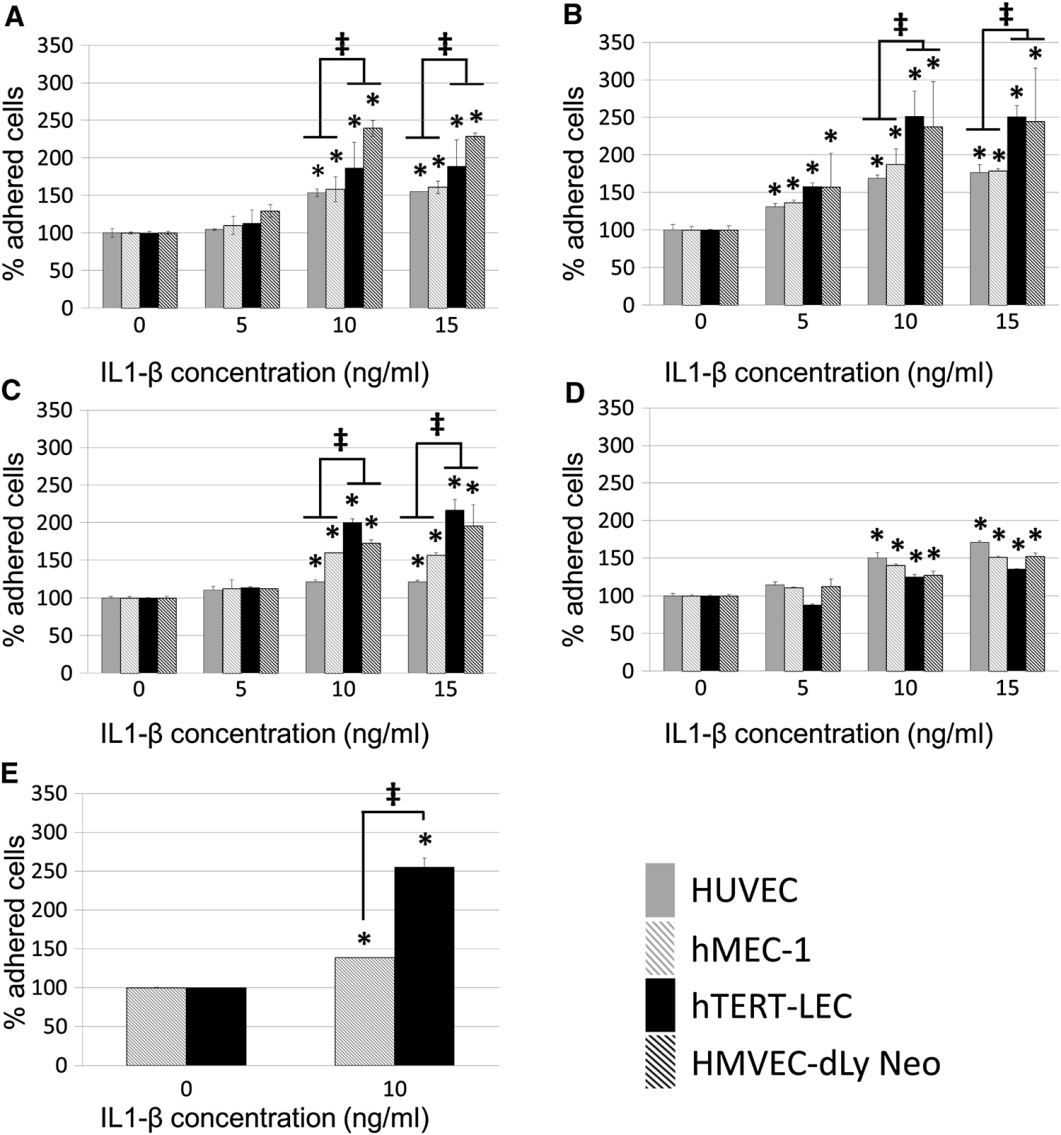
Tumour cell adhesion to blood and lymphatic endothelium with and without IL-1β stimulation. **a** MDA-MB-231 and **b** MCF7 adhesion to IL-1β-stimulated HUVEC (*grey*), hMEC-1 (*grey striped*), hTERT-LEC (*black*) and HMVEC-dLy Neo (*black striped*) endothelial cells. **c** IL-1β stimulated MDA-MB-231 and **d** IL-1β stimulated MCF7 adhesion to unstimulated endothelial cells. **e** IL-1β stimulated MDA-MB-231 adhesion to IL-1β-stimulated hMEC-1 (*grey striped*) or hTERT-LEC (*black*) cells. Statistical significance (*P* < 0.05) determined by *t* test compared to control group is indicated by an *asterisk*, *error bars* represent standard deviation. Statistical significance between blood and lymphatic endothelium is represented by* double dagger*

Conditioning blood or lymphatic endothelial cells with IL-1β for 24 h significantly increased the adhesion of both MDA-MB-231 and MCF7 cells (Fig. 1a, b). Both MDA-MB-231 and MCF7 showed a higher percentage of adhesion to lymphatic endothelial cells than blood endothelial cells following endothelial cell IL-1β stimulation (*P* < 0.001 and *P* < 0.0001, respectively). Adhesion levels of tumour cell lines to endothelial cell models under control conditions have been previously published [19]. As with endothelial cell stimulation, 24-h IL-1β stimulation of tumour cells alone caused significant increase of both MDA-MB-231 and MCF7 adhesion (Fig. 1) to blood and lymphatic endothelial cells with MDA-MB-231 tumour cells having a significantly higher level of adhesion to lymphatic endothelial cells. When both tumour cells and endothelial cells were simultaneously conditioned with IL-1β, adhesion was more markedly increased than when following stimulation of either cell type alone (Fig. 1e).

### Activated macrophages promote tumour cell adhesion to endothelial cells

**Fig. 2:**
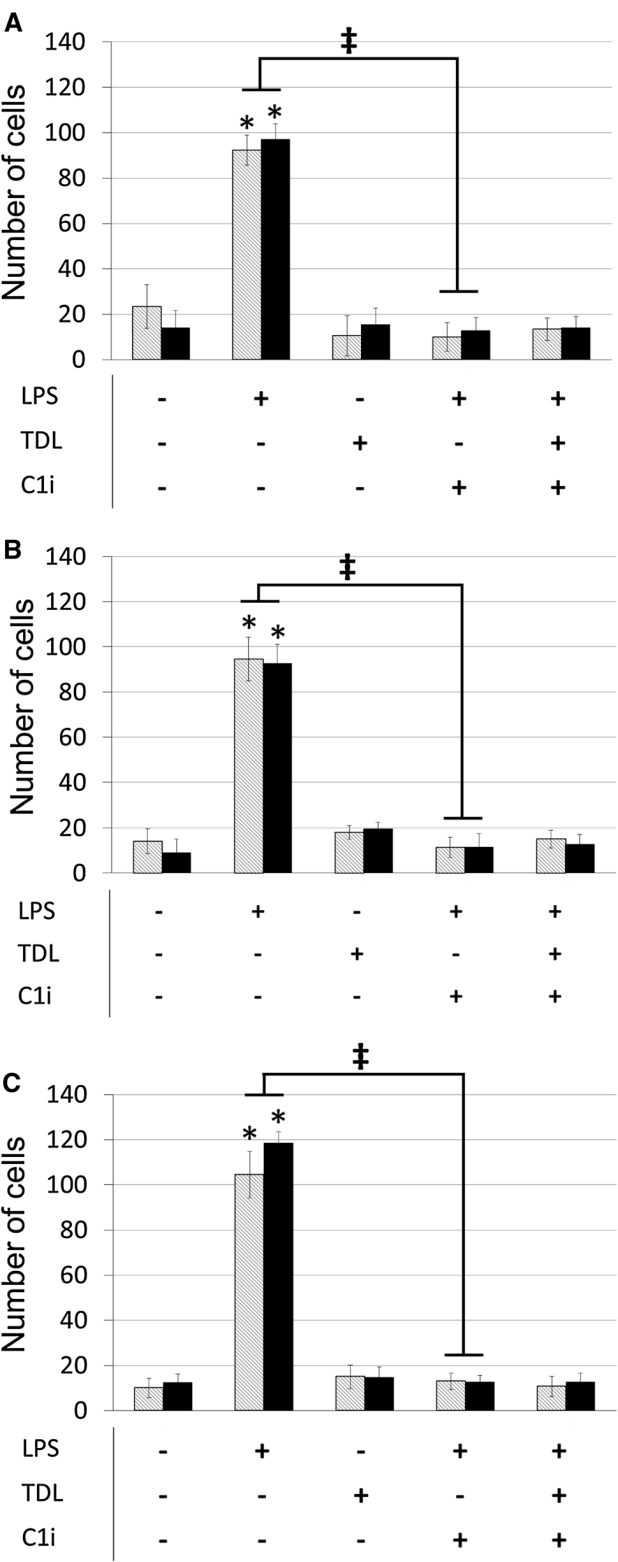
MDA-MB-231 adhesion to macrophage conditioned media stimulated hMEC-1 (*grey striped*) and hTERT-LEC (*black*) from three different donors (**a–c**). Tables indicate macrophage treatment prior to harvesting macrophage conditioned media. *LPS* LPS stimulation, *TDL* tumour-derived lysate stimulation, *C1i* caspase-1 inhibitor. Statistical significance (*P* < 0.05) determined by *t* test compared to control group is indicated by an *asterisk*, *error bars* represent standard deviation. Statistical significance between blood and lymphatic endothelium is represented by* double dagger*

When macrophages from different donors were activated with LPS they secreted IL-1β at varying levels (18–270 pg/ml). Supernatants from LPS activated, but not resting, macrophages markedly increased the adhesion of MDA-MB-231 or MCF7 cells to lymphatic and blood endothelium (Fig. 2a–c). In contrast, tumour conditioned media had no impact on the adhesion of tumour cells to either endothelium (data not shown). Treatment of macrophages with caspase-1 inhibitor reduced secreted IL-1β production by a mean of 73% and concomitantly reduced tumour cell adhesion to levels obtained with resting macrophages (Fig. 2a–c). Activation of macrophages in the presence of tumour-derived lysate also ablated their capacity to promote adhesion to endothelial monolayers.

### IL-1β supports the migratory ability of tumour cells

**Fig. 3:**
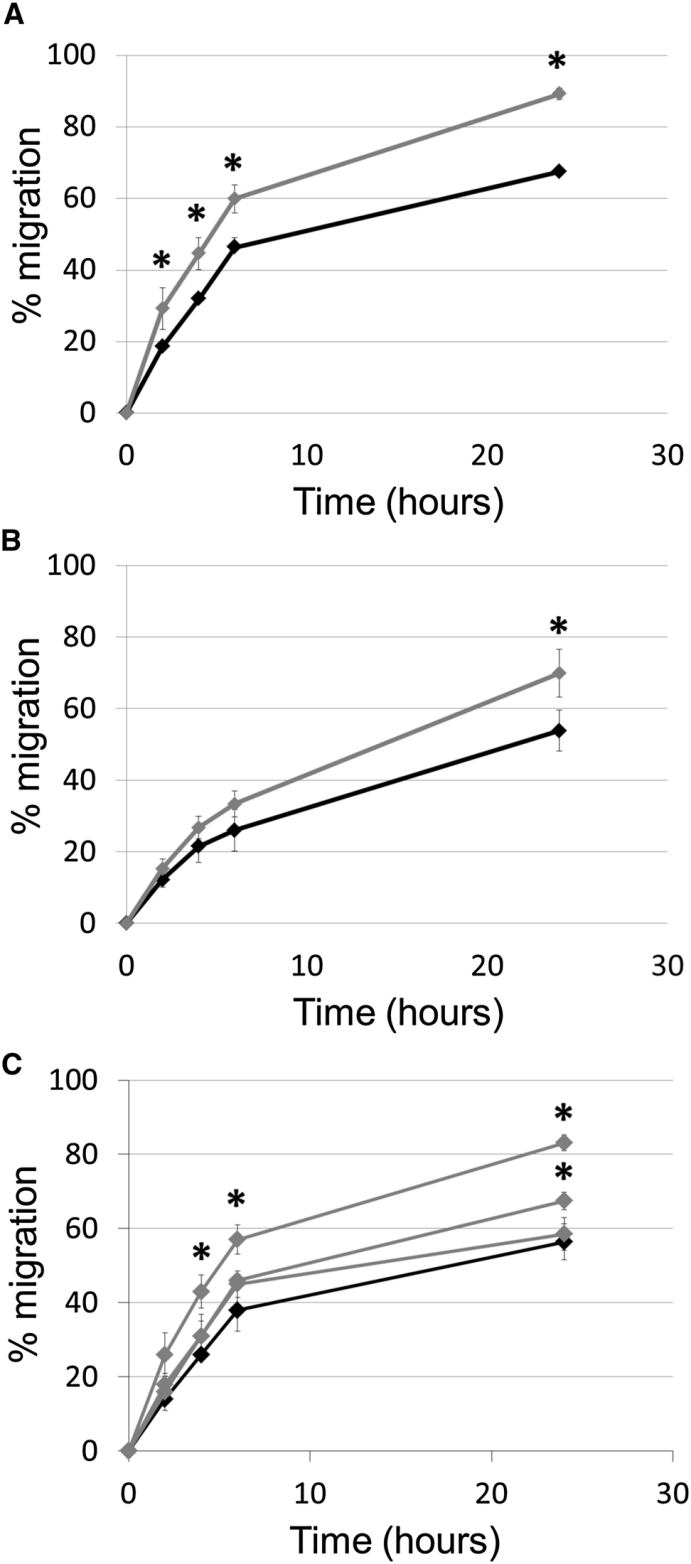
**a** MDA-MB-231 migration in control conditions (*black line*) or following stimulation with IL-1β (*grey line*). **b** MCF7 migration in control conditions (*black line*) or following stimulation with IL-1β (*grey line*). **c** MDA-MB-231 migration in control conditions (*black line*) or following stimulation with macrophage-conditioned media from three individual different donors (*grey lines*). Statistical significance (*P* < 0.05) determined by *t* test compared to control group is indicated by an *asterisk*, *error bars* represent standard deviation

Stimulation of MDA-MB-231 and MCF7 tumour cells with IL-1β caused a significant increase in migration, observed from 2 h in MDA-MB-231 cells and from 24 h in MCF7 cells (Fig. 3a, b). In contrast, tumour-conditioned media did not alter migration of MDA-MB-231 or MCF7 cells (data not shown). Two of the three macrophage-conditioned media significantly increased migration of MDA-MB-231 (Fig. 3c). It was noted that macrophage-derived IL-1β levels in the responding donors were 270 and 137 pg/ml whilst for the non-responding donor there was a much lower level (20 pg/ml). Further analysis showed that the level of IL-1β secreted by activated macrophages corresponded with the rate of tumour cell migration.

**Fig. 4:**
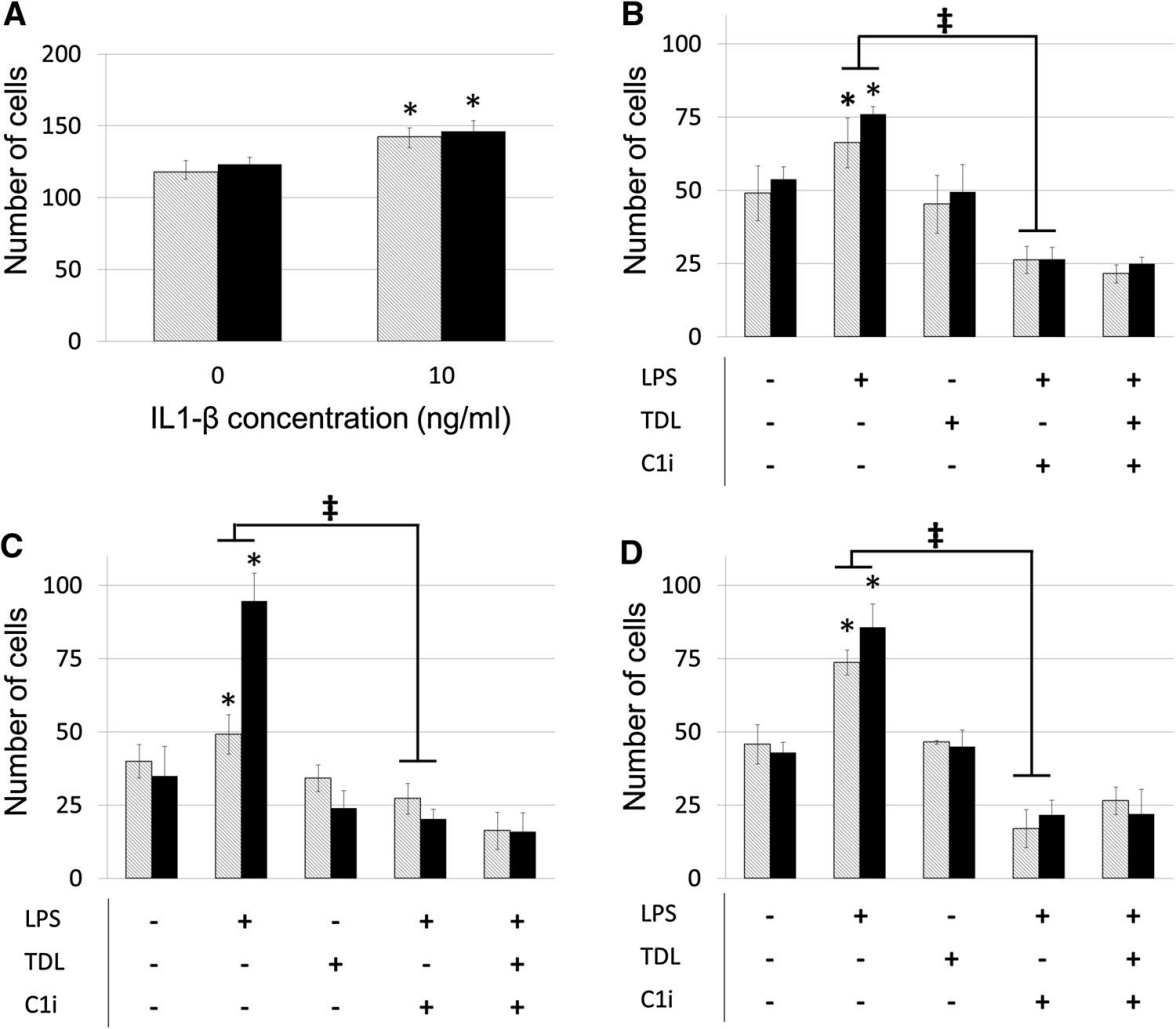
**a** MDA-MB-231 transmigration across hMEC-1 (*grey striped*) and hTERT-LEC (*black*) endothelial cell monolayers under control conditions and following stimulation with IL-1β. **b–d** MDA-MB-231 transmigration across hMEC-1 (*grey striped*) and hTERT-LEC (*black*) endothelial cell monolayers following stimulation with macrophage conditioned media from three individual donors. *LPS* LPS stimulation, *TDL* tumour-derived lysate stimulation, *C1i* caspase-1 inhibitor. Statistical significance (*P* < 0.05) determined by *t* test compared to control group is indicated by an *asterisk*, *error bars* represent standard deviation. Statistical significance between blood and lymphatic endothelium is represented by ‡

We previously established that the primary route of breast tumour metastasis is through lymphatic vessels [2]. We therefore determined the relative capacity of breast tumour cells to traverse blood or lymphatic vessels. A tissue culture model was established using monolayers of blood (hMEC-1) or lymphatic endothelial cells (hTERT-LEC) and the migration of cell lines studied. The addition of IL-1-β to the endothelial monolayer significantly increased tumour cell migration (Fig. 4a). However, there was no preference for migration through lymphatic monolayers. Addition of the conditioned medium from activated macrophages increased the transmigration of MDA-MB-231 cells through both blood and lymphatic endothelial cell barriers (Fig. 4b–d). Importantly, the increased level of transmigration was abrogated by inclusion of a caspase-1 inhibitor.

## Discussion

The aims of this study were to determine the role of IL-1β on adhesion and transmigration to and across endothelial cell monolayers, and whether macrophage might be involved in this process. Studies have shown that lymphatic vessel invasion is more prevalent in patient tumours and is associated with prognosis in numerous tumour types [1, 2].

Following stimulation of endothelial cells with recombinant IL-1β, tumour cell adhesion to blood and lymphatic endothelial cell monolayers increased; however, a larger increase was observed in cells of lymphatic origin. Similar results were observed when MDA-MB-231 cells were stimulated with IL-1β and added to unstimulated endothelial cell monolayers. Interestingly, the preference for MCF7 cells to adhere to lymphatic over blood endothelial cell monolayers when the endothelial cells were stimulated with IL-1β was not replicated when the MCF7 cells were stimulated with IL-1β and added to unstimulated endothelial cells. A substantial increase in MDA-MB-231 adhesion was observed following endothelial cell stimulation with macrophage-conditioned media from stimulated macrophages. Interestingly, dual incubation with LPS and a caspase-1 inhibitor ablated the increase in tumour cell adhesion to endothelial cell monolayers and was associated with a large reduction (62–83%) in the amount of IL-1β present in the macrophage-conditioned media. Tumour-conditioned media had no effect on adhesion and did not contain secreted IL-1β, which is in agreement with previous studies [24].

LPS-stimulated macrophage conditioned media increased transmigration of MDA-MB-231 across both blood and lymphatic endothelium, which could be ablated by including a caspase-1 inhibitor; clearly implicating IL-1β as an important mediator in adhesion and transmigration. Interestingly, in two of three macrophage donors, preferential transmigration across lymphatic endothelium was observed. A study has shown the effect of macrophage conditioned media on MCF7 adhesion to HUVEC which could be reduced with endothelin receptor inhibition and showed similar results for transmigration [25].

We postulate that IL-1β may cause differential expression of adhesion molecules on lymphatic over blood endothelium; we observed an increase of both intracellular adhesion molecule (ICAM)-1 and vascular cell adhesion molecule (VCAM)-1 cell surface expression but to equal levels across HUVEC, hMEC-1 and HTERT-LEC following IL-1β stimulation, with no change in common lymphatic endothelial and vascular endothelial receptor (CLEVER)-1 expression (data not shown). IL-1β has, however, been shown to promote metastasis in a number of tumour types, such as lung cancer [26] and melanoma [14].

In addition to adhesion and transmigration, stimulation of both MDA-MB-231 and MCF7 tumour cells with IL-1β increased their migratory ability; furthermore, this increase was also observed with macrophage conditioned media and could be inhibited with a caspase-1 inhibitor. Previous studies have shown that IL-1β can modulate the migratory potential of MDA-MB-231 cells through accumulation of hypoxia-inducible factor (HIF)-1α, a principal regulator of genes induced by hypoxia [27, 28]. In vivo studies have identified that increased expression of IL-1β is associated with a bone-seeking clone of MDA-MB-231 cells indicating a role for IL-1β in facilitating bone-homing in the process of bone metastasis [29, 30].

The in vitro studies described modelled single phenotypic events and were able to clearly show that IL-1β or macrophage-derived IL-1β enhanced adhesion, migration and transmigration. These data suggest that IL-1β is important for adhesion and transmigration of tumour cells and is likely to be involved in lymphatic vessel invasion.

## Abbreviations

bFGF: Basic fibroblast growth factor
CLEVER-1: Common lymphatic endothelial and vascular endothelial receptor
EBM: Endothelial basal media
EGF: Epidermal growth factor
HIF-1α: Hypoxia-inducible factor
hMEC-1: Human microvascular endothelial cells
HMVEC-dLy Neo: Neonatal dermal lymphatic microvascular endothelial cells
hTERT-LEC: Human telomerase reverse transcriptase immortalised lymphatic endothelial cells
HUVEC: Human umbilical cord vein endothelial cells
ICAM-1: Intracellular adhesion molecule
ICE: IL-1β-converting enzyme
IL: Interleukin
IL-1β: Interleukin-1beta
LPS: Lipopolysaccharide
LVI: Lymphovascular invasion
M-CSF: Macrophage colony stimulating factor
PBMC: Peripheral blood mononuclear cells
STR: Short tandem repeat
TDL: Tumour-derived lysate
TNF: Tumour necrosis factor
VCAM-1: Vascular cell adhesion molecule
VEGF: Vascular endothelial growth factor
